# Ultrasound Back-Fat Thickness Association with Risk of Metabolic Disease of Dairy Cows in Early Lactation

**DOI:** 10.3390/ani15060883

**Published:** 2025-03-20

**Authors:** Filippo Fiore, Enrico Fiore, Barbara Contiero, Anastasia Lisuzzo

**Affiliations:** 1 Department of Veterinary Medicine, University of Sassari, 07100 Sassari, Italy; 2 Department of Animal Medicine, Production, and Health (MAPS), University of Padua, 35020 Legnaro, Italy; enrico.fiore@unipd.it (E.F.); barbara.contiero@unipd.it (B.C.); anastasia.lisuzzo@unipd.it (A.L.)

**Keywords:** back-fat thickness, hyperketonemia, body-condition score, ultrasonography, dairy cows

## Abstract

Ultrasound back-fat thickness (BFT) can indirectly assess the risk of metabolic disease as hyperketonemia. In this study, 129 multiparous and clinically healthy dairy cows were enrolled and examined weekly for the first 8 weeks of lactation for body-condition score (BCS), BFT, β-hydroxybutyrate (BHB), weekly changes (ΔBCS, ΔBFT, ΔBHB), and total changes over the study (TotalΔ_8to1_BCS, TotalΔ_8to1_BFT, TotalΔ_8to1_BHB). Cows with BHB ≥ 1.0 mmol/L were considered to be affected by hyperketonemia (HK; n = 56), while the remaining animals were considered as controls (CTR; n = 73). Statistical analysis included mixed models, spearman correlation matrix, logistic regression, and linear regression analysis. BCS and BFT showed a strong correlation and were greater in the first weeks after calving in HK. The same group had greater BCS and BFT losses over the trial. However, weekly changes were identified only for BFT. Linear and logistic regression analysis for the disease event identified that BFT loss of 1 mm was associated with an increase in BHB (+0.36 mmol/L) and an increased risk of developing hyperketonemia over the week when the loss was between the second and first weeks (+2.5 times), third and second (+51%), and fourth and third (+58%) weeks. In conclusion, BFT can be used to identify the risk of developing hyperketonemia during early lactation.

## Simple Summary

Subclinical ketosis or hyperketonemia can significantly impact animals’ health. The body condition score is a subjective method associated with hyperketonemia. On the contrary, ultrasound measurements of back fat thickness are an objective method that could assess the risk of metabolic disease. A total of 129 multiparous dairy cows were followed weekly during the first 8 weeks after calving with both methods and checked for hyperketonemia. Two groups were established retrospectively: control or healthy animals (n = 73), and diseased animals (n = 56). Our results showed that the body-condition score and back-fat thickness were strongly related. Moreover, animals with greater body-condition score or back-fat thickness at the beginning of lactation had a higher risk of developing hyperketonemia as well as animals with greater losses over the postpartum period. Moreover, the risk of developing hyperketonemia was greater with 1 point of body-condition score loss compared to the loss of 1 mm of back-fact thickness. However, the results suggest that the decrease in back-fat thickness could be easily identified over weeks contrasting with body-condition score. In conclusion, the back-fat thickness could represent a useful method to assess the hyperketonemia risk during the postpartum period.

## 1. Introduction

The imbalance between energy intake and requirements after calving induces a para-physiological negative energy balance in dairy ruminants during early lactation. Body resources as muscle, adipose, and bone tissues are then mobilized to support colostrum and milk production [1]. This mobilization predisposes animals to many disorders as dystocia, retained placenta, metritis, reduced feed intake after calving, mastitis, displaced abomasum, fatty liver syndrome, and ketosis [2,3,4]. Moreover, all these diseases are interrelated and negatively impact animals’ health and productivity, and are a consequence of high metabolic rates around calving [2,3,4].

Ketosis was classified according to clinical signs into clinical and subclinical ketosis. As suggested by the terminology itself, clinical ketosis represents an increase in ketone bodies (acetoacetate, acetone, and β-hydroxybutyrate or BHB) above a certain threshold value associated with clinical signs [5,6]. In contrast, no clinic signs are present in subclinical ketosis, in which the increase in ketone bodies remains. Subclinical ketosis was over time also referred to as hyperketonemia, which represents precisely this increase in the concentration of ketone bodies above values considered physiological and does not describe a clinical presentation [5,6]. Usually, the BHB is used to diagnose hyperketonemia with a blood threshold of 1.0 mmol/L [7]. Hyperketonemia can significantly impact animals’ health due to the increased risk of other diseases such as abomasum dislocation, reproduction disorders, and infectious disease, which reduces milk production and increases the risk of culling [8,9].

The mobilization of adipose tissue consequent to negative energy balance is the reason for the changes in the body-condition score (BCS). The BCS is an estimation of the body reserves amount in animals though the investigation of such areas as the spinal column, spinous processes, ribs, hips, tuber ischia, tail head, and thigh region [10]. The scoring system of this parameter usually involves a 5-point scale established in the United States (1 to 5 with 3 as ideal BCS) for dairy cows [10]. Other BCS scales have been developed over time: four points for dairy cow scales adapted from a rank used in beef cattle; six points proposed in the UK; eight points proposed in Australia; and 10 points introduced in New Zealand [10]. Despite the used scoring system, the high values of BCS (over-conditioned animals) at calving are associated with lower postpartum dry matter intake (DMI), greater loss of post-partum BCS, and a greater incidence of metabolic disorders, particularly ketosis, hypocalcemia, abomasal dislocation, and hepatic lipidosis [11,12]. Considering these associations and its easy application, the BCS method has become a key tool in the management of dairy herds [13]. However, the BCS is a subjective method, and variations as small as about 0.25 points may not be correctly identified.

The back-fat thickness or BFT is a non-invasive, more accurate, and objective method to assess subcutaneous fat in contrast to BCS. Subcutaneous fat can be used to estimate adipose mass and, consequently, energy balance of animals [14,15]. Thereby, this method is less susceptible to classifier bias compared to the BCS, which leads to higher comparability across studies, and BFT losses can be used to indirectly assess the negative energy balance and the risk of metabolic disease [15,16]. The BFT measurement can be taken at the dorsal level between 12th and 13th rib for a better association with carcass characteristics, generally in beef cattle, or it can be taken at the level of the sacral region for a better association with BCS, generally in dairy cattle [14,17].

For this reason, the aim of this study was to evaluate the association between BFT and hyperketonemia to identify an objective method for a field assessment of hyperketonemia risk.

## 2. Materials and Methods

### 2.1. Animals and Study Design

The experimental study was evaluated and approved by the Ethics Committee of the University of Sassari with the following approval number: 50677/2018.

The study was carried out for 1 year in three commercial dairy farms in the north of Sardinia (Italy) during regular herd supervision activity carried out by the veterinary practitioner. Each farm consisted of approximately 200 lactating Holstein Friesian dairy cows housed in free-stall barns and milked twice a day. The total mixed ratio (TMR) met or exceeded National Research Council requirements for 650-kg lactating cows producing 35 kg/day of milk with 3.5% fat and 3.1% protein [18].

A total of 248 multiparous Holstein Friesian dairy cows calved in the study period. Among these, animals with clinical disease during the study period (mastitis, claw disorder, dystocia, retained placenta, metritis, and displaced abomasum) were excluded. Consequently, 129 Holstein Friesian dairy cows equally divided in the three farms were enrolled in the study with a parity of 2 (n = 25, 19.4%), 3 (n = 36, 27.9%), 4 (n = 37, 28.7%), and from ≥5 (n = 31, 24.0%). Enrolled animals were investigated by veterinary practitioners during gynecological activities once a week for the first 8 weeks after calving for BCS, BFT, and BHB.

### 2.2. The Body-Condition Score (BCS) and Back-Fat Thickness (BFT)

**Figure 1:**
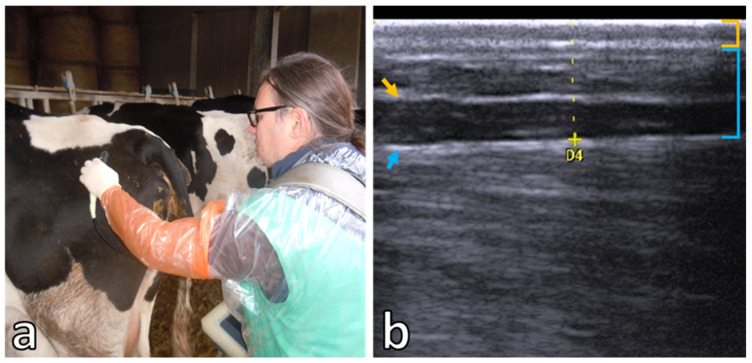
(**a**) Example of ultrasound probe placement for the back-fat thickness (BFT) assessment. (**b**) Ultrasound scan for the measurement of BFT (dashed yellow line). Orange arrow indicates fascia trunci superficialis; blue arrow indicates fascia trunci profunda; orange square bracket indicates skin; and blue square bracket indicates subcutaneous fat.

The BCS was evaluated using the five-point visual BCS technique with 0.25 increment as proposed by Edmonson et al. [19]. The BFT was measured with a real-time, B-mode diagnostic ultrasound scanner equipped with a 5.0 MHz linear transducer probe (Esaote MyLab™Touch-Vet, SV3513 VET Array 10-5 Mhz; Esaote, Genova, Italy) after the application of coupling gel as a transducing agent. The settings were maintained constant with a frequency of 10 MHz, a 7 cm depth acoustics window, and a100% gray scale gain. Time-gain compensation was in a neutral position. The probe was placed vertically to an imaginary line between the pins (*tuber ischia*) and hooks (*tuber coaxe*) at the sacral examination site (≈9–11 cm cranial to the pins; Figure 1a) [15]. Images were saved in a digital imaging and communications in medicine (DICOM) format and used for post-sampling quantitative assessment (MyLabDesk^TM^, Esaote S.p.a., Genova, Italy). The BFT represents the thickness (mm) of subcutaneous fat between the skin and the *fascia trunci profunda* located above the *gluteus medius* muscle (Figure 1b) [15]. Both BCS and BFT evaluation was assessed by the same vet practitioner with experience and training in assessment of BCS and ultrasound measurement.

The BCS and BFT variations (ΔBCS and ΔBFT) among each time point were used to assess changes (loss or gain) in energy reserves. Specifically, the data of each time point was lowered by the value of the previous evaluation with the exception of the week 1 after calving, which was without previous evaluation. The total post-calving variations in BCS and BFT were measured using the last evaluation at week 8 after calving, lowered by the baseline value of week 1 after calving (Total Δ_8to1_ BCS and Total Δ_8to1_ BFT).

### 2.3. The β-Hydroxybutyrate (BHB) and Hyperketonemia Cases

Blood sampling was collected from the coccygeal vein after morning milking but before feeding through a 5-mL syringe and was immediately evaluated for BHB concentration by a portable digital reader (Abbott Precision Xtra™ meter, Oxon, UK) and blood ketone test strips (Abbott Precision Xtra™ Blood Ketone test strips, Oxon, UK). The BHB variation (ΔBHB) of each time point was lowered by the value of the previous evaluation with the exception of the week 1 after calving, which was without previous evaluation. The total post-calving variations in BHB were measured using the last evaluation at week 8 after calving, lowered by the baseline value of week 1 after calving (Total Δ_8to1_ BHB).

Cows with BHB ≥ 1.0 mmol/L were considered to be affected by hyperketonemia [7,20], and they were treated with 250 mL of propylene glycol two times per day for 3 days (Glicole propilenico FU-USP; A.C.E.F. s.p.a., Fiorenzuola d’Arda (PC), Italy). Animals with at least one case of hyperketonemia during the study period were enrolled in the Hyperketonemia group (HK, n = 56). Remaining animals that displayed a BHB < 1.0 mmol/L at each time point were enrolled in the Control group (CTR, n = 73).

An animal that showed high BHB levels (≥ 1.0 mmol/L) separated by one or more sampling time points with healthy levels (BHB < 1.0 mmol/L) was considered as a new case of hyperketonemia for the same animal. Hyperketonemia prevalence (%; n. hyperketonemia cases/n. enrolled animals) and incidence (%; n. new hyperketonemia cases/(n. new hyperketonemia cases + n. healthy animals)) were estimated at each time point (from week 1 to week 8). Furthermore, cumulative incidence (%) was measured for the study period.

### 2.4. Statistical Analysis

Differences over time in hyperketonemia prevalence (%) and incidence (%) were evaluated with the Chi-square test by the MedCalc software ver. 19.4 (MedCalc Software, Ostend, Belgium).

Statistical analysis of the parameters under evaluation (BCS, BFT, BHB, ΔBCS, ΔBFT, ΔBHB, Total Δ_8to1_ BCS, Total Δ_8to1_ BFT, and Total Δ_8to1_ BHB) was conducted with R software version 4.2.3 (https://www.R-project.org/; accessed on 27 November 2024). The BCS, ΔBCS, and Total Δ_8to1_ BCS showed a non-normal distribution at the Shapiro–Wilk test, and they were analyzed by two unpaired sample Wilcoxon tests for group comparison (HK vs. CTR), and Kruskal–Wallis test associated with Dunn Test for changes over time within group. The BFT, BHB, ΔBFT, and ΔBHB were analyzed by mixed model with fixed effect of group, week, their interaction, and the random and repeated effect of the animal. A similar model without the fixed effect of week was used for Total Δ_8to1_ BFT and Total Δ_8to1_ BHB. In both cases, a post hoc pairwise comparison among least squares means was performed using Bonferroni correction. The *p*-value accepted as significant was ≤ 0.05. A Spearman correlation analysis was applied to two matrixes: i. BCS, BFT, BHB, ΔBCS, ΔBFT, and ΔBHB; ii. Total Δ_8to1_ BCS, Total Δ_8to1_ BFT, and Total Δ_8to1_ BHB.

Linear regression analysis was conducted with PROC REG of the S.A.S. system software (version 9.4; SAS Institute Inc., Cary, NC, USA) using the BHB as a dependent variable, and BCS, BFT, ΔBCS, and ΔBFT as independent variables. Two logistic regression analyses were conducted with PROC GLIMMIX of the same software using the effect of group (HK vs. CTR) as a dichotomous outcome variable, and disease event (week of hyperketonemia case per animal without reference to the group to which it belongs) as predictors to estimate the odds ratio (OR) with the 95% of confidence interval (95%CI). The *p*-value accepted as significant was ≤0.05.

## 3. Results

Animals were sampled on average at 4.6 ± 1.8 days in milk (DIM) for week 1, 11.5 ± 2.1 DIM for week 2, 18.4 ± 2.2 DIM for week 3, 25.0 ± 2.9 DIM for week 4, 32.2 ± 2.4 DIM for week 5, 39.1 ± 2.4 DIM for week 6, 46.0 ± 2.7 DIM for week 7, and 53.1 ± 2.9 DIM for week 8.

A total of 56 animals had at least one case of hyperketonemia (blood BHB ≥ 1.0 mmol/L) during the study period, and they were enrolled in the HK group. Among these animals, 60.7% (n = 34) was identified as diseased for only 1 week, 21.4% (n = 12) for 2 weeks, 12.5% (n = 7) for 3 weeks, 3.6% (n = 2) for 4 weeks, and 1.8% (n = 1) for 8 weeks. Among animals recognized as diseased for 1 week, 23.5% (n = 8) had two hyperketonemia cases, and 8.8% (n = 3) had three hyperketonemia cases. Among animals recognized as diseased for 2 and 3 weeks, 16.7% (n = 2) and 42.9% (n = 3) showed a second hyperketonemia case, respectively.

**Figure 2:**
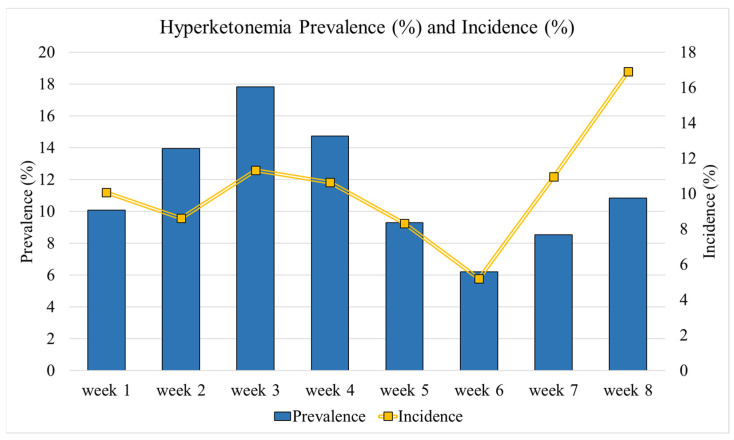
Hyperketonemia prevalence (%) and incidence (%) from week 1 to week 8 after calving.

Hyperketonemia prevalence and incidence were presented in Figure 2. The mean hyperketonemia prevalence was 11.4% with the highest prevalence at week 3 (17.8%, n = 23) and the lowest at week 6 (6.2%, n = 8). On the contrary, the highest hyperketonemia incidence was at week 8 (16.9%, n = 11) and the lowest was at week 6 (5.2%, n = 4). The cumulative incidence for the 8 weeks of the study period was 50.7%.

**Figure 3:**
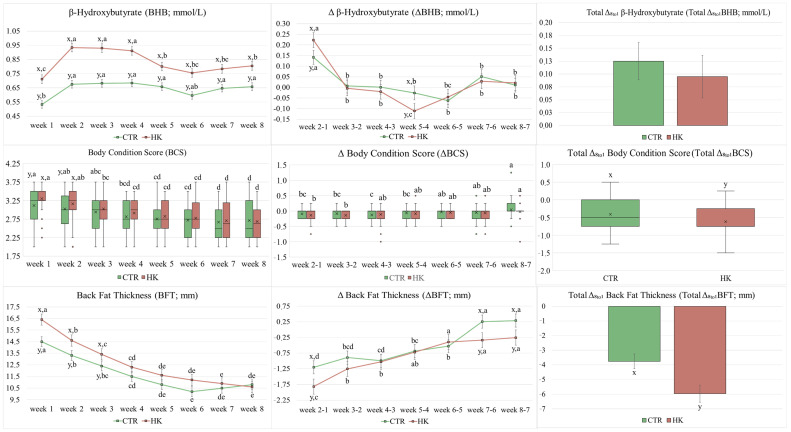
The body-condition score (BCS), back-fat thickness (BFT; mm), β-hydroxybutyrate (BHB; mmol/L), ΔBCS, ΔBFT, ΔBHB, Total Δ_8to1_ BCS, Total Δ_8to1_ BFT, and Total Δ_8to1_ BHB for Hyperketonemia group (HK, n = 56) and Control group (CTR, n = 73) over time. ^a–e^ Significant differences over time within group; ^x–y^ Significant differences between group within time point.

Figure 3 displays the results of the parameters under evaluation (BCS, BFT, BHB, ΔBCS, ΔBFT, ΔBHB, Total Δ_8to1_ BCS, Total Δ_8to1_ BFT, and Total Δ_8to1_ BHB). The BHB and ΔBHB showed a significant effect of the interaction between group and week (*p* = 0.017 and *p* = 0.013). For the first parameter, it was always highest in the HK group compared with the CTR group with a peak between week 2 and week 4. The latter parameter was greatest in the HK group (greater BHB increment) compared to the CTR group at week 2 versus week 1 (week 2-1), while it was lowest in HK group (greater BHB reduction) compared to the CTR group at week 5 versus week 4 (week 5-4). However, the Total Δ_8to1_ BHB was not significant for group effect (*p* = 0.586). The BCS had significant differences over time for the CTR group (*p* < 0.001) and for the HK group (*p* < 0.001), while a significant difference between groups was evidenced at week 1 (*p* = 0.010) and week 2 (*p* = 0.042) with generally greater values in the HK group. The ΔBCS was significant over time (*p* < 0.001 for both the CTR and the HK group), but no differences were identified between groups. Instead, the Total Δ_8to1_ BCS was lowest in the HK group (greater BCS reduction; *p* = 0.005). The BFT (*p* = 0.006) and ΔBFT (*p* = 0.046) were significantly influenced by group and week. The BFT was greatest in the HK group compared to the CTR group at week 1, week 2, and week 3. The ΔBFT was lowest in the HK group (greater BFT reduction) compared to the CTR group at week 2 versus week 1 (week 2-1), week 7 versus week 6 (week 7-6), and week 8 versus week 7 (week 8-7). Moreover, also the Total Δ_8to1_BFT was lowest in the HK group (greater BFT reduction) compared to the CTR group (*p* = 0.005).

**Figure 4:**
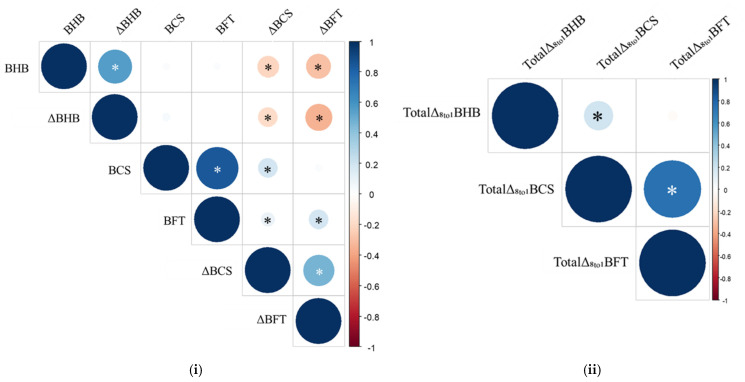
A Spearman correlation analysis of the two matrixes: (**i**). BCS, BFT, BHB, ΔBCS, ΔBFT, and ΔBHB; and (**ii**). Total Δ_8to1_ BCS, Total Δ_8to1_ BFT, and Total Δ_8to1_ BHB. Both white and black (*) indicated a significant correlation (*p* ≤ 0.05).

The Spearman correlation matrixes (Figure 4) displayed:

▪ two strong correlations between BCS and BFT (r = 85.1%, *p* < 0.001), and between Total Δ_8to1_ BCS and Total Δ_8to1_ BFT (r = 74.7%, *p* < 0.001)
▪ two moderate correlations between BHB and ΔBHB (r = 49.7%, *p* < 0.001), and between ΔBCS and ΔBFT (r = 44.5%, *p* < 0.001)
▪ Eight weak correlations between ΔBFT and ΔBHB (r = −33.3%, *p* < 0.001), BHB and ΔBFT (r = −26.3%, *p* < 0.001), BCS and ΔBCS (r = 17%, *p* < 0.001), Total Δ_8to1_ BCS and Total Δ_8to1_ BHB (r = 15.2%, *p* = 0.006), BHB and ΔBCS (r = −15%, *p* < 0.001), BFT and ΔBFT (r = 14.5%, *p* < 0.001), ΔBCS and ΔBHB (r = −12.3%, *p* < 0.001), and between BFT and ΔBCS (r = 6.9%, *p* = 0.047).

The linear regression analysis highlighted a significant effect of ΔBCS and ΔBFT (*p* < 0.001 for both) on BHB variations. Specifically, the BHB increase of 0.21 ± 0.04 mmol/L for each point of BCS reduction, and BHB increase of 0.36 ± 0.01 mmol/L for each mm of BFT reduction.

**Figure 5:**
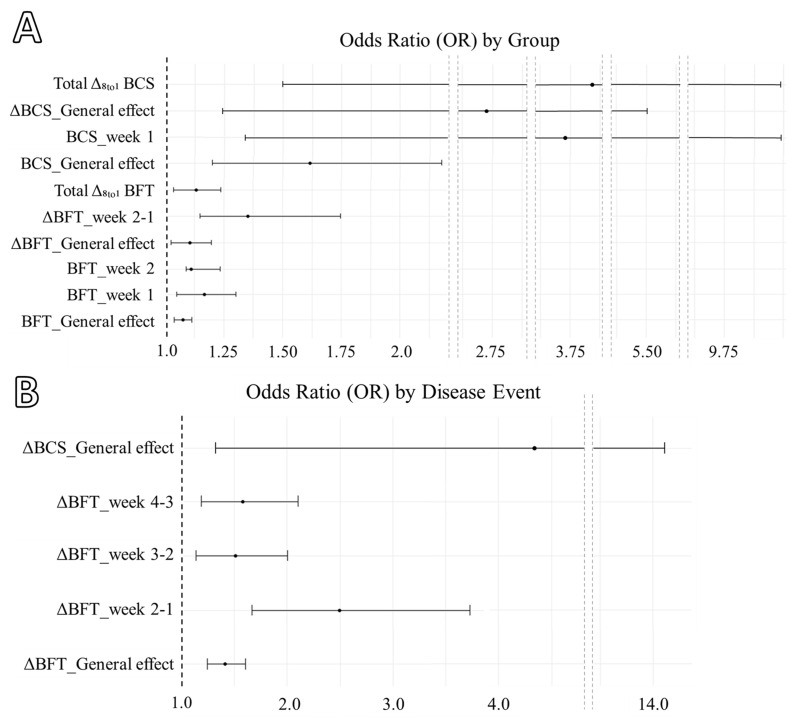
Forest plot of the significant odds ratio (OR) with 95% of confidence interval by Group (HK vs. CTR; (**A**)) and by disease week event (**B**).

The logistic regression analysis based on the group effect (HK vs. CTR; Figure 5A) showed a significant effect for BFT (*p* < 0.001), BCS (*p* = 0.002), ΔBFT (*p* = 0.011), ΔBCS (*p* = 0.012), Total Δ_8to1_ BCS (*p* = 0.005), and Total Δ_8to1_ BFT (*p* = 0.007). Specifically, +1 mm of BFT was generally associated with an increased risk to develop hyperketonemia during the 8 weeks after calving (OR: 1.07; 95%CI: 1.04 to 1.11), especially in week 1 (OR: 1.17; 95%CI: 1.05 to 1.30) and week 2 (OR: 1.11; 95%CI: 1.00 to 1.23). Similarly, a +1-point of BCS generally increased the risk of hyperketonemia (OR: 1.62; 95%CI: 1.20 to 2.18), especially in week 1 (OR: 3.62; 95%CI: 1.33 to 9.89). Regarding the variations over weeks (ΔBFT and ΔBCS), the loss of 1 mm of BFT generally increased the disease risk (OR: 1.11; 95%CI: 1.02 to 1.20) especially between week 2 and week 1 (week 2-1; OR: 1.35; 95%CI: 1.15 to 1.75). Instead, the loss of one point of BCS generally increased the risk (OR: 2.62; 95%CI: 1.24 to 5.54) without a specific risk among weeks. The loss of one point of BCS and 1 mm of BFT during the 8 postpartum weeks (Total Δ_8to1_ BCS and Total Δ_8to1_ BFT) increased the hyperketonemia risk (OR: 3.86 and 1.13; 95%CI: 1.51 to 9.90 and 1.04 to 1.24).

The logistic regression analysis based on the disease event (Figure 5B) showed a significant effect for ΔBFT (*p* < 0.001) and ΔBCS (*p* = 0.015). Specifically, the loss of 1 mm of BFT increased the risk to develop hyperketonemia during the examined week (OR: 1.41; 95%CI: 1.24 to 1.60), especially if the examined week was week 2-1 (OR: 2.50; 95%CI: 1.67 to 3.73), week 3-2 (OR: 1.51; 95%CI: 1.14 to 2.00), and week 4-3 (OR: 1.58; 95%CI: 1.18 to 2.11). Regarding the ΔBCS, the loss of one point increased the risk for the examined week (OR: 4.35; 95%CI: 1.34 to 14.2).

## 4. Discussion

Propylene glycol is a treatment options for clinical and subclinical ketosis due to the contribution in oxaloacetate level via pyruvate or lactate routes. However, its effects are often transient after treatment [6], as also shown in the present study. In fact, 17.9% of the animals showed a hyperketonemia condition for 3 or more weeks despite the treatment, and several animals (28.6%) had two or more hyperketonemia cases. These results highlighted that simply supporting glucose metabolism is reductive for the treatment of ketosis. In fact, this disease has several types of deficiencies as amino acids and vitamins that should be taken into account when treating animals [7,21].

The negative energy balance usually reaches the nadir in the first 10 DIM and can last up to 30–100 DIM. The 75% of disease, including ketosis, occur in the first month postpartum [3]. The prevalence of this disease ranges from 11.2% to 47.2% for subclinical ketosis or hyperketonemia, and from 3.7% to 11.6% for clinical ketosis. In contrast, the incidence ranges from 19.7% to 44% for hyperketonemia, and it is about 2.4% for clinical ketosis [22,23]. Furthermore, the cumulative hyperketonemia incidence for the first 30 DIMs is about 40% [24]. In our study, the results were similar to the literature. In fact, the mean prevalence was 11.4%, with the highest value at week 3 (17.8%) and the lowest at week 6 (6.2%). In contrast, the incidence ranged from 5.2% (week 6) to 16.9% (week 8), with a cumulative incidence of 50.7% for the first 8 weeks postpartum. These findings further suggest how hyperketonemia can have a major impact on animal health and well-being since it tended to affect about one in two animals during the first 8 weeks of lactation.

The BHB level of the HK group was consistently greater than that of the CTR group during the study period. However, the average BHB value was always less than 1.0 mmol/L. This result is due to the different distribution of the prevalence and incidence of hyperketonemia during the trial. Regarding week-to-week changes (ΔBHB), the HK group showed a greater increase between the second and first week (week 2-1) than the CTR group. The changes between weeks 3 and 2 (week 3-2), as well as between weeks 4 and 3 (week 4-3), were less marked than the previous one. In addition, the HK group showed a greater decrease in ΔBHB between week 5 and 4 (week 5-4). These results agreed with the plateau phase of the BHB level consistent with the high level of the hyperketonemia prevalence. In addition, it is interesting to note that the BFT had a strong reduction in the weeks with BHB plateau (week 1 to week 5), while the BCS presented a marginal difference. In fact, a negative correlation, although weak, was shown between BHB and ΔBFT, and between ΔBHB and ΔBFT.

Generally, the optimal BCS at calving is between 3.0 to 3.5 (five-point scale) with a maximum loss of 0.5 to 1.0 points in 40–100 DIM (BCS at nadir 2.50 to 2.75) [10]. This indication was maintained for the animals in this study with a median BCS of 3.25 for both groups at week 1, and a median of 2.50 and 2.63 at week 8 for the CTR and HK groups, respectively. However, the HK group had generally greater BCS values than the CTR group in the first 2 weeks after calving. Total BCS loss (Total Δ_8to1_ BCS) was within the recommended and above-mentioned range but was higher in the HK group (median loss: −0.50 and −0.75 for the CTR and HK groups). Despite the Total Δ_8to1_ BCS change differed, no differences were found between the two groups in weekly BCS losses (ΔBCS). In fact, BCS changes of 0.25 points (five-points scale) may not be detected between two consecutive assessments even when performed by experienced operators [14,25]. Moreover, this small variation is still difficult to identify even when animals are examined three weeks after calving [26].

Excessive conditioning of dry dairy cows is known to be a risk factor for metabolic diseases, especially hyperketonemia and hepatic lipidosis. This risk is found to be particularly higher when greater BCS is present in the first 2 weeks after calving [14,15,26]. In fact, animals with greater BCS at calving tend to have lower DMI during postpartum, causing a worsening negative energy balance and an increased lipomobilization (greater BCS loss). The increased lipomobilization of non-esterified fatty acids (NEFAs) tends to increase hepatic lipid concentration and further worsens DMI, thus creating a self-perpetuating cycle [27,28]. In this study, the risk for the animal suffering of at least one hyperketonemia case in the first 8 weeks after calving appeared to be particularly important when considering the overall BCS effect and its value in the first week after calving. In particular, the risk increased by +62% and +3.6 times, respectively, for each additional point of BCS in line with what has been reported in the literature. However, the BCS value did not affect the hyperketonemia risk when considering the week in which the disease occurred and was diagnosed (disease event). Consequently, this result suggests how the animals’ management and nutrition during the dry period was essential to reduce the hyperketonemia risk during the postpartum period. However, the BCS assessment was not sufficient to identify the risk of developing hyperketonemia during the week of the animal’s observation.

As well as over-conditioning animals, excessive lipomobilization also increases the hyperketonemia risk [15]. Animals that lose more than 0.75 points of BCS have from 4.6 to 5.0 times an increased risk of developing subclinical ketosis, as well as an increased risk of reproductive problems (abortion, placenta retention, and metritis), mastitis, hypocalcemia, and lameness [13,29]. In general, severe lipomobilization is identified with a loss greater than or equal to 0.02 BCS point per day, equivalent to one point in 60 days [30]. Both weekly and total BCS loss (ΔBCS and Total Δ_8to1_ BCS) also influenced the risk of developing hyperketonemia during postpartum in our study. In general, a weekly one-point loss in BCS increased the risk by 2.6 times in our study, while the risk increased by 3.9 times with a one-point loss between week 1 and week 8. The ΔBCS variation also increased the risk of developing hyperketonemia during the week of observation (disease event) by 4.4 times when animal lost one point of BCS compared with the previous week. However, animals generally lose maximum one point of BCS in 40–100 DIM, as reported above, with the greatest lost within 30 DIM. Consequently, important changes between two consecutive weeks are not common. Furthermore, small changes in BCS (around 0.25-points) are unlikely found between consecutive weeks [15,16,26]. Therefore, the risk assessment by BCS loss, although extremely impactful, appears to be unrealistic for routinely animals’ management and nutrition to prevent hyperketonemia.

The BFT represents an objective and highly repeatable evaluation of adipose tissue reserves and their mobilization as opposed to the BCS [15]. Moreover, these two parameters are usually well-correlated with values between 35% and 87% [15,31]. A strong correlation between BCS and BFT was also shown in our study and in line with literature (r = 85.1%), which it was confirmed also in total and weekly losses of both parameters. Similar to BCS, the dairy cows of the HK group exhibited greater BFT values in the first 3 weeks after calving suggesting that a greater BFT at calving negatively affects animal health increasing the risk of hyperketonemia during the post-partum such as with BCS. In fact, the risk of developing hyperketonemia over the study period increases by +7% for each additional mm of BFT, with an even higher risk in the first (week 1; +17%) and second weeks (week 2; +11%) after calving.

The energy balance of dairy cows usually reaches a positive condition by 45 DIM, and 90% reach the positive balance by 63 DIM [26]. The BFT nadir can be variable and ranges from 50 to 125 DIM, with a loss between 7 and 10 mm [14,16]. In fact, the study of Siachos et al. [31] reports an average loss of 3.6 mm of BFT in 28 days after calving. Generally, the BFT loss can range from −0.08 to −0.18 mm/d until 50 DIM [14]. In addition, cows with metabolic diseases such as ketosis and/or over-conditioned cows tend to lose more BFT during postpartum [13,27]. Thereafter, this parameter tends to increase particularly between the third and second month of lactation [25]. The HK group showed a greater BFT loss between the second and first week after calving (ΔBFT week 2-1; 1.8 mm for HK vs. 1.21 mm for CTR). In addition, the same group continued to show a BFT loss at week 7-6 (46.1 DIM) and week 8-7 (53.2 DIM) in contrast to the CTR group, which began to increase in BFT. Consequently, the Total Δ_8to1_ BFT loss was greater in the HK group than in the CTR group (−6.0 mm or −0.75 mm/week vs. −3.8 mm or −0.48 mm/week). These results agreed with the literature for the greater BFT reduction in diseased animals. Furthermore, they suggest that animals with at least one case of hyperketonemia in the postpartum period had a longer BFT loss, and thus potentially also a longer negative energy balance, over the post-calving period than healthy animals.

Excessive lipomobilization and ketosis were identified by a BFT loss of 10 mm in 60 days (−1.17 mm/week) [30]. Elevated BFT losses have been associated with higher levels of BHB, NEFA, and AST [32,33]. In our study, the HK group showed a lower BFT loss per week (generally around −0.75 mm/week) and in contrast with literature. Moreover, the linear regression analysis identified an increase in BHB level by +0.36 mmol/L for each mm of BFT loss. The hyperketonemia risk during postpartum was found to be increased by +11% per each mm of BFT loss, especially between the first 2 weeks (week 2-1) with an increased risk of +35%. The Total Δ_8to1_ BFT change also indicated a +13% increased risk for each mm of BFT loss between week 8 and week 1. In contrast, the risk of developing the disease during the week of observation (disease event) was found to be +41% per each mm of BFT loss compared to the previous week, especially at week 2-1 +2.5 times), week 3-2 (+51%), and week 4-3 (+58%). These results suggest that constant monitoring of BFT, even during routinely gynecological activities of vets, allows for a more objective and applicable identification of the hyperketonemia risk both considering the risk to develop hyperketonemia during the first 8 weeks after calving (group effect), and considering the risk to develop hyperketonemia during the week of observation compared to the previous week (disease event).

## 5. Conclusions

The results of this study showed that greater BFT at the beginning of lactation increases the risk of hyperketonemia as well as BCS. However, BCS variation over the week could not be useful to recognize hyperketonemia risk in field activities, considering that very small changes (about 0.25 points) in the latter parameter are difficult to identify and the loss of 1.0 point or more are uncommon. In contrast, small changes in BFT can be identified by an objective method allowing a clear definition of the risk of developing hyperketonemia. This risk was found to be +2.5 times when 1 mm of BFT was lost between the second and first week after calving, +51% between the third and second week, and +58% between the fourth and third week. Finally, the animals with hyperketonemia had a longer BFT loss in contrast to the control group. In conclusion, the BFT could represent a useful, easily applicable, and more accurate method in-field for an indirect evaluation of the risk of developing metabolic diseases after calving. Further studies, including various facilities and dairy breeds, could be useful to identify the hyperketonemia risk in dairy cows experiencing different production systems.

## Data Availability

The raw data supporting the conclusions of this article will be made available by the corresponding authors on request.
